# Distribution of arbuscular mycorrhizal fungi (AMF) in Terceira and São Miguel Islands (Azores)

**DOI:** 10.3897/BDJ.8.e49759

**Published:** 2020-04-01

**Authors:** Catarina Drumonde Melo, Christopher Walker, Helena Freitas, Artur Câmara Machado, Paulo A. V. Borges

**Affiliations:** 1 cE3c – Centre for Ecology, Evolution and Environmental Changes / Azorean Biodiversity Group and Universidade dos Açores - Departamento de Ciências Agrárias e do Ambiente, Rua Capitão João d’Ávila, São Pedro, 9700-042, Angra do Heroísmo, Terceira, Azores, Portugal cE3c – Centre for Ecology, Evolution and Environmental Changes / Azorean Biodiversity Group and Universidade dos Açores - Departamento de Ciências Agrárias e do Ambiente, Rua Capitão João d’Ávila, São Pedro, 9700-042 Angra do Heroísmo, Terceira, Azores Portugal; 2 CFE - Centre for Functional Ecology, Department of Life Sciences, University of Coimbra, 3001-401, Coimbra, Portugal CFE - Centre for Functional Ecology, Department of Life Sciences, University of Coimbra, 3001-401 Coimbra Portugal; 3 Royal Botanic Garden Edinburgh, 20A Inverleith Row, EH3 5LR, Edinburgh, United Kingdom Royal Botanic Garden Edinburgh, 20A Inverleith Row, EH3 5LR Edinburgh United Kingdom; 4 School of Agriculture and Environment, University of Western Australia, 35 Stirling Highway, Perth WA 6009, Crawley, Australia School of Agriculture and Environment, University of Western Australia, 35 Stirling Highway, Perth WA 6009 Crawley Australia; 5 CFE – Centre for FunctionalCFE - Centre for Functional Ecology, Department of Life Sciences, University of Coimbra, 3001-401, Coimbra, Portugal CFE – Centre for FunctionalCFE - Centre for Functional Ecology, Department of Life Sciences, University of Coimbra, 3001-401 Coimbra Portugal; 6 CBA-UAç – Biotechnology Center of Azores, Universidade dos Açores - Departamento de Ciências e Engenharia do Ambiente, Rua Capitão D´Ávila, 9700-042, Angra do Heroísmo, Portugal CBA-UAç – Biotechnology Center of Azores, Universidade dos Açores - Departamento de Ciências e Engenharia do Ambiente, Rua Capitão D´Ávila, 9700-042 Angra do Heroísmo Portugal

**Keywords:** Arbuscular mycorrhizal fungi (AMF), native forest, *Juniperus brevifolia*, *Picconia azorica*, semi-natural and intensive pastures

## Abstract

**Background:**

The data, presented here, come from samples collected during three research projects which aimed to assess the impact of land-use type on Arbuscular Mycorrhizal Fungi (AMF) diversity and community composition in pastures of Terceira Island (Azores, Macaronesia, Portugal) and also in the native forest of two Azorean Islands (Terceira and São Miguel; Azores, Macaronesia, Portugal). Both projects contributed to improving the knowledge of AMF community structure at both local and regional scales.

**New information:**

Little is known on the AMF communities from Azores islands and this study reports the first survey in two Azorean Islands (Terceira and São Miguel). A total of 18,733 glomeromycotan spores were classified at the species level from 244 field soil samples collected in three different habitat types – native forests (dominated by *Juniperus brevifolia* and *Picconia azorica*), semi-natural and intensively-managed pastures. Thirty-seven distinct spore morphotypes, representing ten glomeromycotan families, were detected. Species of the family *Acaulosporaceae* dominated the samples, with 13 species (38% of the taxa), followed by *Glomeraceae* (6 spp.), *Diversisporaceae* (4 spp.), *Archaeosporaceae* (3 spp.), *Claroideoglomeraceae* (3 spp.), *Gigasporaceae* (3 spp.), *Ambisporaceae* and *Paraglomeraceae*, both with the same number of AMF species (2 spp.), *Sacculosporaceae* (1 sp.) and *Entrophospora* (family insertae sedis). Members of the family *Acaulosporaceae* occurred almost exclusively in the native forests especially associated with the *Picconia azorica* rhizosphere, while members of *Gigasporaceae* family showed a high tendency to occupy the semi-natural pastures and the native forests of *Picconia azorica*. Members of *Glomeraceae* family were broadly distributed by all types of habitat which confirm the high ecological plasticity of this AMF family to occupy the more diverse habitats.

## Introduction

Arbuscular mycorrhizal fungi (AMF) are one of the most important groups of below-ground biota (Jeffries et al. 2003; Barea et al. 2005). These obligate symbionts live in association with approximately 80% of vascular plants and have essential ecological roles, namely, they facilitate plant growth through enhancing uptake of several macro- and micro-nutrients of low mobility (e.g. P, Zn, Cu) in soil (Brundrett and Tedersoo 2018). Arbuscular mycorrhizas can also provide other ecological functions such as influencing the microbial and chemical environment of the mycorrhizosphere, stabilising soil aggregates (Rillig and Mummey 2006) and conferring plant tolerance to several abiotic (Göhre and Paszkowski 2006; Li et al. 2013; Chitarra et al. 2016) and biotic (Vos et al. 2012; van der Heijden et al. 2015) stresses.

AMF are, therefore, beneficial for plant performance, playing a crucial role for the sustainability of natural and agricultural ecosystems (Barea et al. 2011) and important ecosystem services (Chen et al. 2018). However, despite their ecological role, little is known about how their community structure varies in relation to habitat type in the Azores archipelago.

The Azores archipelago has an extended area of grasslands (Martins 1993), including natural grasslands, semi-natural pastures and intensive pastures (Cardoso et al. 2009). It also has the unique native forest, Laurisilva, which has more endemic plants and animals than any other habitat in the region. In the last 500 years, as a consequence of human activity, much of this native forest has been replaced by man-made habitats and has been subjected to fragmentation (Borges et al. 2005). Thus, immediate action to restore and expand native forest is required to avoid the ongoing loss of endemic species (Terzopoulou et al. 2015). AMF play an important role in habitat restoration, by improving plant nutrition and performance under environmental stress by facilitating plant adaptation in both nursery and field conditions (Babu and Reddy 2011). Therefore, understanding the AMF diversity in the native forest will help to define strategies for management and restoration of such endangered forests. An important step in restoration strategies is the re-establishment of adapted native plant species (Ferrol et al. 2004). A good understanding of mycorrhizal associations in undisturbed localities could then be used to provide information about AMF inoculum production for use in the rehabilitation of degraded ecosystems.

In this contribution, we list the species of Arbuscular Mycorrhizal Fungi (AMF) found in ecological studies, comparing anthropogenically disturbed pastures and forests of Terceira Island (Azores, Macaronesia, Portugal) and also in the native forests of São Miguel Island (Azores, Macaronesia, Portugal).

## General description

### Purpose

In this contribution, we list the AMF species found in pastures from different land-use types of Terceira Island to investigate the effect of disturbance on AMF community structure. Native forests from Terceira and São Miguel Island were also sampled to observe patterns of AMF species composition and distribution, in order to provide baseline information for later use in establishing strategies for conservation of *Picconia azorica* and *Juniperus brevifolia*, in particular and native Azorean forests, in general.

## Project description

### Study area description

**Figure 1.**
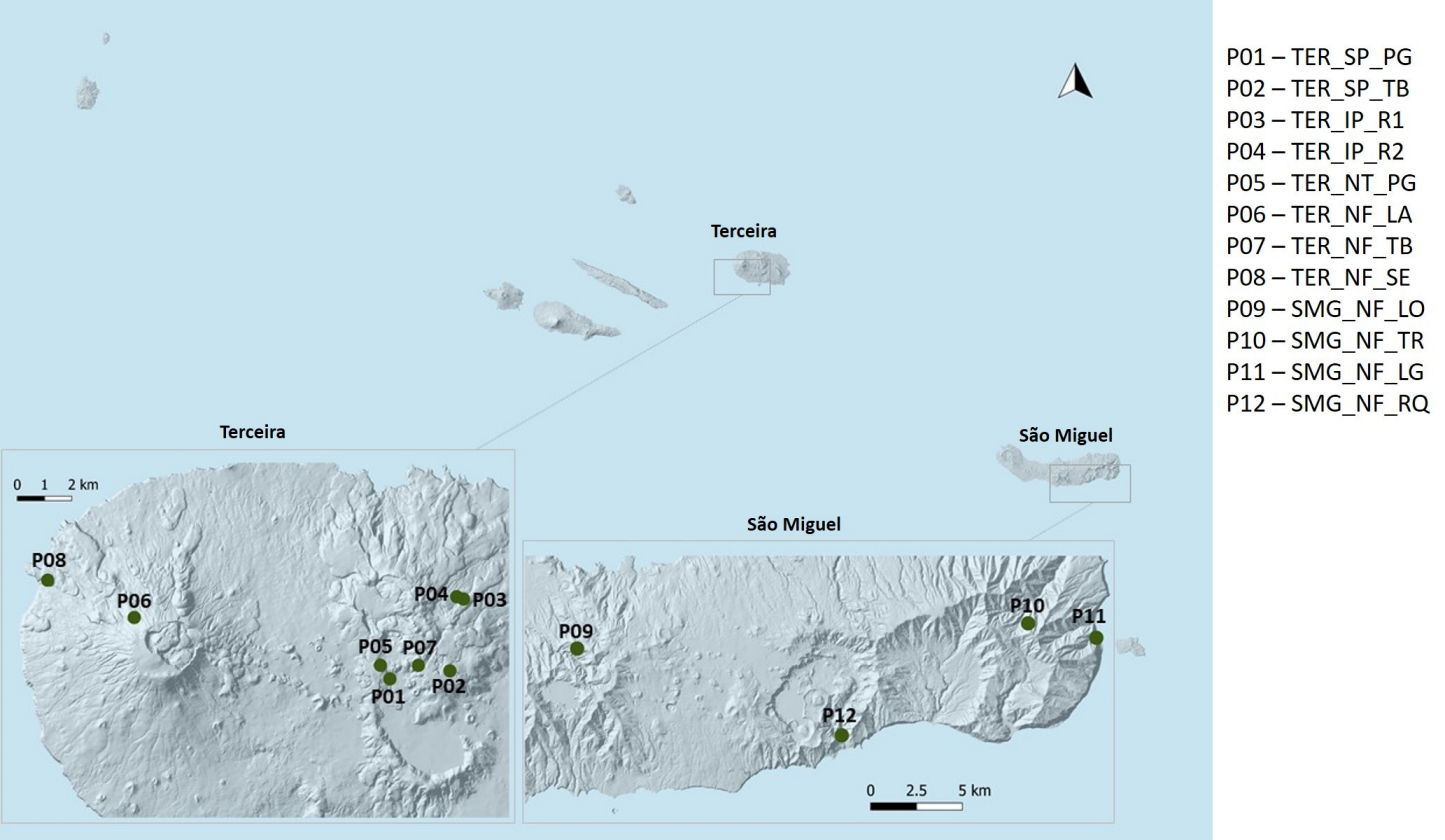
Location of sampling sites in Terceira and São Miguel Islands (Azores).

**Figure 2.**
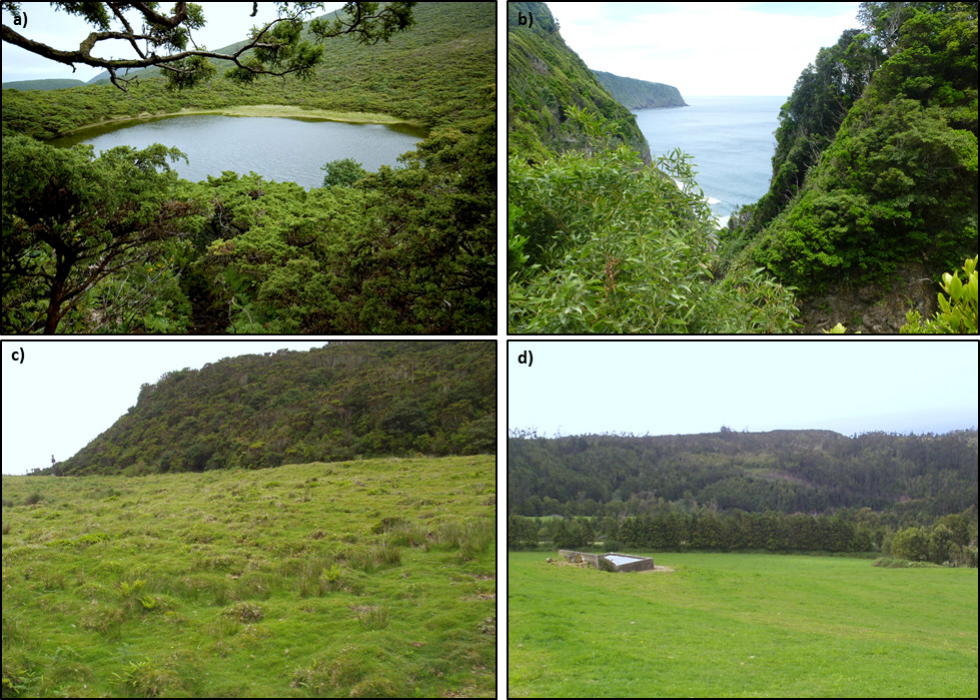
Habitat types in Terceira and São Miguel Islands, Azores: **a.** native forest of *Juniperus brevifolia*; **b.** native forest of *Picconia azorica*; **c.** semi-natural pastures; **d.** intensively-managed pastures.

All data used in this study came from surveys about AMF diversity and composition in different ecosystems (pasturelands and native forests) conducted in two Islands of the Azorean archipelago, Terceira and São Miguel (Melo et al. 2014, Melo et al. 2017, Melo et al. 2018) (Fig. 1). The sampling areas were cattle-grazed upland pastures of two different types from Terceira and four fragments of native forests from each Island (Table 1) (Fig. 2). The two pasture types include semi-natural pastures with low grazing intensity and frequency (managed for more than 50 years, with low stocking density, grazed only in summer and with a relatively high diversity of grasses and forbs) and intensively-managed pastures with high grazing intensity and frequency (managed for more than 30 years, with high stocking density, grazing during all year and characterised also by a depauperate vascular flora of five or fewer dominant species) (Melo et al. 2014). The semi-natural pastures, Pico Galhardo (TER_SP_PG) and Terra Brava (TER_SP_TB) (Fig. 1) are included in Terceira Natural Park and are dominated by the perennial grasses *Holcus lanatus* and *Agrostis castellana*, have a high floristic diversity (Dias 1996, Borges 1997), often including other grasses such as *Anthoxanthum odoratum*, *Lolium multiflorum*, *Holcus rigidus* and *Poa trivialis* and non-forage species including *Lotus uliginosus*, Rumex acetosella ssp. angiocarpus, *Potentilla anglica*, *Hydrocotyle vulgaris*, *Plantago lanceolata and Lobelia urens* (For more details see Melo et al. 2014). The intensively-managed pastures, Agualva 1 (TER_IP_R1) and Agualva 2 (TER_IP_R2) (Fig. 1) resulted from the conversion of undisturbed native forest to wood production of non-native trees and then to permanent pastures. They are now surrounded by an exotic eucalyptus plantation. The vegetation is dominated by *Holcus lanatus* and *Lolium perenne*, but also has high populations of *Trifolium repens*, *P. lanceolata*, *Cyperus esculentus*, *Mentha suaveolens*, *Cerastium fontanum* and *Rumex conglomeratus (Dias 1996, Borges 1997*).

In Terceira Island, the native forests included two fragments from Natural Park – Pico Galhardo (TER_NF_PG) and Lagoinha (TER_NF_LA) (Fig. 1) (Melo et al. 2017), both dominated by the Azorean cedar *Juniperus brevifolia*, a rare conifer species that is endemic to Azores, which dominates at high-elevation (> 650 m), with subdominant endemic woody perennials, including *Laurus azorica* (Lauraceae), *Ilex perado azorica* (Aquifoliaceae), *Erica azorica* (Ericaceae), *Vaccinium cylindraceum* (Ericaceae) and *Frangula azorica* (Rhamnaceae) (Elias et al. 2016). Nevertheless, in Lagoinha, invasive woody species, including *Cryptomeria japonica*, *Pittosporum undulatum* (Pittosporaceae), *Eucalyptus globules* and *Acacia melanoxylon* (Fabaceae), have begun to establish. The remaining two native fragments from Terceira Island include two populations of *Picconia azorica* – Terra Brava (TER_NF_TB) and Serreta (TER_NF_SE) (Fig. 1). Terra Brava is located in the very wet Laurisilva at 650 m altitude (Fig. 1) being dominated by endemic woody plants, predominantly *L. azorica*, *I. azorica*, *Frangula azorica*, *V. cylindraceum*, *E. azorica*, *Myrsine africana* and, occasionally, by *J. brevifolia* and *P. azorica*. Serreta (NFSE) is located at low altitude (95 m) (Fig. 1) and is characterised by a low diversity of plants, dominated by *Morella faya* and *P. azorica* and, occasionally, by *L. azorica.* These forests are located in the most thermophilic areas of Azores and are almost extinct (Dias 1996). The highest canopy is dominated by a dense cover of *P. undulatum* and, rarely, by *L. azorica*. This forest is mixed with other invasive woody species, including *Metrosideros excelsa*, *E. globules*, *A. melanoxylon*, *Sphaeropteris cooperi*, *Fuchsia magellanica* and *Rubus inermis*. The herbaceous stratum is dominated by *Dryopteris azorica*, Hedera helix var. azorica, *Smilax aspera* and *Gomphocarpus fruticosus* (Dias 1996).

In São Miguel Island, the four native fragments are two populations of *J. brevifolia* up to 700 m altitude (Lombadas and Tronqueira) and two populations of *P. azorica* in the lowlands around 95 m altitude in Lombo Gordo and Ribeira Quente (Fig. 1). Lombadas (SMG_NF_LO) is included in the Natural Reserve of Lagoa do Fogo in São Miguel (Fig. 1). Although the surrounding vegetation is dominated by the introduced species *C. japonica*, *Clethra arborea*, *A. melanoxylon* and *E. globulus*, this forest still retains several endemic elements, including *J. brevifolia*, *V. cylindraceum* (Ericaceae), *L. azorica*, *Euphorbia stygiana* (Euphorbiaceae), *F. azorica*, *E. azorica*, *I. azorica* and *Culcita macrocarpa* (Culcitaceae) (Silva 2001). Tronqueira (SMG_NF_TR) is located in hyperhumid native forest (Fig. 1), a type of forest that has been largely replaced by other land uses (Moreira et al. 2012), resulting in an abundance of exotic plants, such as *C. japonica* and *C. arborea*. The tree layer is composed of the endemic woody plants *J. brevifolia*, *I. azorica*, *L. azorica*, *M. africana* (Myrsinaceae) and *E. azorica*, while the shrub layer is mostly formed by *V. cylindraceum* and *Viburnum treleasei* (Adoxaceae). Lombo Gordo (SMG_NF_LG) is covered by a coastal scrubland (Fig. 1) where *P. azorica* dominates in certain areas, but is mixed with other native and invasive woody species including *M. faya*, *E. azorica*, *P. undulatum*, *Arundo donax*, *Hedychium gardnerianum* and *Phormium tenax* (Martins et al. 2011). Ribeira Quente (SMG_NF_RQ) is also a coastal scrubland (Fig. 1) dominated by the endemic plants *L. azorica* and *P. azorica* but also associated with other native and invasive woody species, such as *P. undulatum*, *M. faya* and *A. melanoxylon*.

### Design description

Arbuscular mycorrhizal fungi (AMF) diversity and composition were investigated at three habitat types: native forests of *J. brevifolia* and *P. azorica*, semi-natural pastures and intensively-managed pastures. Each habitat type was represented by two sites. At each site from semi-natural (Pico Galhardo (10.39 ha) – TER_SP_PG; Terra Brava (8.81 ha) – TER_SP_TB) and intensively (Agualva 1 (5.03 ha) – TER_IP_R1; Agualva 2 (3.06 ha) – TER_IP_R2) managed pastures, ten soil samples were collected in August 2007 (i.e. a total of 40 soil samples) (Project CD_Melo_PhD). In natural forests of *J. brevifolia* from Terceira (Pico Galhardo (13.97 ha) – TER_NF_PG; Lagoinha (3.05 ha) – TER_NF_LA) and from São Miguel (Lombadas (37.42 ha) – SMG_NF_LO; Tronqueira (51.70 ha) – SMG_NF_TR), 21 soil samples were collected from seven marked *J. brevifolia* plants in each site at three different sampling times (September 2012; May 2013; September 2013) in both islands, resulting in a total of 84 soil samples (2 islands × 2 sites/island × 7 samples/site × 3 sampling dates) (CD_Melo_Postdoc; FCT - PTDC /AGR-ALI/122152/2010). In natural forests of *P. azorica* from Terceira (Terra Brava (9.72 ha) - TER_NF_TB; Serreta (7.67 ha) – TER_NF_SE) and from São Miguel (Lombo Gordo (41.69 ha –SMG_ NF_LG; Ribeira Quente (6.32 ha) – SMG_NF_RQ), 30 soil samples were collected from ten marked *P. azorica* plants in each site, during the same sampling times for *J. brevifolia*, in both islands resulting in 120 soil samples (2 islands × 2 sites/island × 10 samples/site × 3 sampling dates) (CD_Melo_Postdoc; FCT - PTDC /AGR-ALI/122152/2010).

## Sampling methods

### Sampling description

In semi-natural and intensively-managed pastures, the soil samples with associated roots were randomly collected with a shovel, from the rooting zone of the dominant plant species, *H. lanatus*, to a depth of 0 - 20 cm. In native fragments of *P. azorica*, the distance between samples taken on each site was a minimum of 25 m and maximum of 40 m and the distance between sample sites was about 20 km in Terceira and 15 km in São Miguel. Each soil sample was geo-referenced and consisted of four subsamples collected from different points (approximately N, S, E and W) around the rooting zone of each *P. azorica* plant with a shovel to a depth of 0 - 20 cm or 0 - 30 cm, depending on the soil conditions and the depth of rhizosphere system. The litter layer was removed during sampling and replaced afterwards. Subsequent samples were taken from the same marked plants following the cardinal points. In the case of native fragments of *J. brevifolia*, the distance between samples taken on each site was between 25 m and 40 m and the distance between sample sites was about 5 km in Terceira and 24 km in São Miguel. The sample collection followed the same procedure as for *P. azorica* (Melo et al. 2017). For all habitat types, each soil sample consisted of approximately 2 kg of rhizosphere soil. In the lab, the soil samples were air-dried, sieved through a 2 mm mesh and stored at 4ºC before analysis.

### Quality control

Frequently, spores directly extracted from the soil are low in number and contaminated by other organisms, which makes their identification difficult. Consequently, it is necessary to establish trap cultures to promote sporulation and provide specimens for detailed examination. Open pot-trap cultures (Gilmore 1968) were established from each soil sample collected at semi-natural and intensive pastures with one-week-old *Zea mays* seedlings (Melo et al. 2014). Soil samples collected from the native forests were used to establish two of such cultures, one with one-week-old *Z. mays* seedlings and another one with micropropagated *J. brevifolia* and *P. azorica* seedlings (Melo et al. 2017, Melo et al. 2018). Establishment of single or multi-spore cultures of the different AM fungal morphotypes with *Plantago lanceolata* as host plant was attempted in pots of river sand. Spores with a healthy appearance (oily contents; without evidence of contamination by non-AMF) of each AM fungal morphotype were used as inoculum by placing them on a seedling root system under a dissecting microscope, immediately before transplanting into the pot (Melo et al. 2017, Melo et al. 2018). Specimens were given a voucher number, linked to their culture attempt number. Individual microscope slides were numbered serially so that photographic images could be traced back to their specimen of origin and details were recorded in a database to allow complete tracking of culturing history and linkage of related voucher specimens. The new cultures were placed in a climate-controlled plant growth chamber. When needed, cultures were watered with deionised water. Individuals of morphologically characterised spore types, extracted from field soil, trap cultures or single spore cultures, were used for DNA analysis. Molecular characterisation, including DNA extraction, PCR, cloning, RFLP, sequencing and phylogenetic analyses, is published in Melo et al. (Melo et al. 2017, Melo et al. 2018).

### Step description

Glomeromycotan spores were extracted from 50 g of air-dried soil from each sample (field soil, trap cultures and single or multi-spore cultures) by wet sieving and sucrose centrifugation (Walker 1992) and stored at 4ºC in autoclaved water, pending examination. Different spore types were initially separated in water under a stereomicroscope. Representatives of each morphotype were identified through a compound microscope in a 4:1 mixture of polyvinyl alcohol lacto-glycerol (PVLG) and Melzer’s reagent, photographed and stored as semi-permanent slide preparations. Counts were made for the total number of spores of each morphotype under a dissecting microscope after classification into either known species or types that could not be placed in a current species, based on colour, size, surface ornamentation, hyphal attachment, reaction to Melzer’s reagent and wall structure. Identification of spores was carried out by use of primary literature and experience from more than 40 years of taxonomic study of the Glomeromycota by C. Walker (e.g., Walker and Trappe 1981, Walker et al. 1984, Koske and Walker 1985, Walker et al. 1986, Walker and Diederichs 1989, Walker 1992, Walker and Vestberg 1998, Schüßler et al. 2011, Krüger et al. 2012, Redecker et al. 2013, Walker et al. 2018, Wijayawardene et al. 2018, Schüßler and Walker 2019) and joint authorship with A. Schüßler of the website amf-phylogeny.com, which lists all accepted species in the phylum. The illustrated manual of Blaszkowski (2012) was also used. Comparisons were made, where possible, with the type and the authenticated specimens and with other literature such as original species descriptions, websites (e.g. http://invam.wvu.edu) and examination of other well-documented specimens, when available.

## Geographic coverage

### Description

Terceira and São Miguel Islands, the Azores, Macaronesia, Portugal.

### Coordinates

37.423 and 38.959 Latitude; -27.532 and -24.917 Longitude.

## Taxonomic coverage

### Taxa included

**Table:** 

Rank	Scientific Name	Common Name
phylum	Glomeromycota	Arbuscular mycorrhizal fungi

## Collection data

### Collection name

AMF data base

### Collection identifier

Catarina Melo

### Parent collection identifier

Christopher Walker

### Specimen preservation method

PVLG-Melzer slide

## Usage rights

### Use license

Creative Commons Public Domain Waiver (CC-Zero)

## Data resources

### Data package title

Distribution of Arbuscular Mycorrhizal Fungi in Terceira and S. Miguel (Azores, Portugal)

### Resource link

http://ipt.gbif.pt/ipt/resource?r=arbuscular_mycorrhizal_fungi_terceira_azores

### Alternative identifiers

https://www.gbif.org/dataset/c72a7a97-9de0-4854-aa80-90df6389ff12

### Number of data sets

1

### Data set 1.

#### Data set name

arbuscular_mycorrhizal_fungi_terceira_azores

#### Data format

Darwin Core Archive

#### Number of columns

43

#### Download URL

http://ipt.gbif.pt/ipt/resource?r=arbuscular_mycorrhizal_fungi_terceira_azores

#### Data format version

1.4

#### Description

The following data table includes all the records for which a taxonomic determination of the species was possible. The dataset submitted to GBIF (Melo et al. 2019) is structured as a sample event dataset, with two tables: event (as core) and occurrences. The data in this sampling event resource have been published as a Darwin Core Archive (DwCA), which is a standardised format for sharing biodiversity data as a set of one or more data tables. The core data table contains 226 records (eventID). One extension data table also exists with 665 occurrences. An extension record supplies extra information about a core record. The number of records in each extension data table is illustrated in the IPT link. This IPT link archives the data and thus serves as the data repository. The data and resource metadata are available for downloading in the downloads section.

**Data set 1.** 

Column label	Column description
Table of Events	Table with sampling events data
eventID	Identifier of the events, unique for the dataset
samplingProtocol	The sampling protocol used to capture the species
eventRemarks	Remarks of the plant species from where the specimens were extracted
sampleSizeValue	The numeric amount of time spent in each sampling
sampleSizeUnit	The unit of the sample size value
eventDate	Date or date range when the record was collected
Year	Year of the event
Month	Month of the event
country	Country of the sampling site
locality	Name of the locality
stateProvince	Name of the region of the sampling site
island	Name of the island
locationID	Identifier of the location
habitat	The surveyed habitat
DecimalLatitude	Approximate centre point decimal latitude of the field site in GPS coordinates
DecimalLongitude	Approximate centre point decimal longitude of the field site in GPS coordinates
coordinateUncertaintyInMetres	Uncertainty of the coordinates
coordinatePrecision	Precision of the coordinates
georeferenceSources	Method used to obtain coordinates
eventRemarks	The list of Projects supporting the sampling event
Table of Occurrences	Table with species density data (beginning of new table)
CatalogNumber	Unique identification code for species density data
eventID	Identifier of the events, unique for the dataset
occurrenceID	Identifier of the record, coded as a global unique identifier
licence	Reference to the licence under which the record is published
institutionID	The identity of the institution publishing the data
institutionCode	The code of the institution publishing the data
basisOfRecord	The nature of the data record
kingdom	Kingdom name
phylum	Phylum name
class	Class name
order	Order name
family	Family name
genus	Genus name
specificEpithet	Specific epithet
scientificNameAuthorship	The authorship information for the scientificName formatted according to the conventions of the applicable nomenclaturalCode
scientificName	Complete scientific name including author
taxonRank	Lowest taxonomic rank of the record
organismQuantity	A number or enumeration value for the quantity of organisms
organismQuantityType	The unit of the identification of the organisms
identifiedBy	Name of the person who made the identification
occurrenceRemarks	DwC associatedSequence - A list (concatenated and separated) of identifiers (publication, global unique identifier, URI) of genetic sequence information associated with the occurrence

## Additional information

A total of 53,208 glomeromycotan spores, representing 97 distinct morphotypes, were classified from 244 field soil samples. However, only 37, including 18,733 spores, could be classified morphologically at the species level, eight of which were also characterised by molecular methods (Tables 2, 3). The families with most AMF species were *Acaulosporaceae* (13 spp.), followed by *Glomeraceae* (6 spp.), *Diversisporaceae* (4 spp.) and *Archaeosporaceae*, *Claroideoglomeraceae* and *Gigasporaceae*, all with 3 spp. Spores from the *Acaulosporaceae* were found in almost all samples (85%), followed by those from *Gigasporaceae* (48%), *Glomeraceae* (39%), *Archaeosporaceae* (18%) and *Claroideoglomeraceae* (13%). Of the 37 AMF identified, 18 AMF occurred only in Terceira (49%), most of which were from *Acaulosporaceae*, 17 AMF were found in both Islands (46%) and just two from the *Paraglomeraceae* and *Sacculosporaceae*, respectively only occurred in São Miguel (5%).

**Figure 3.**
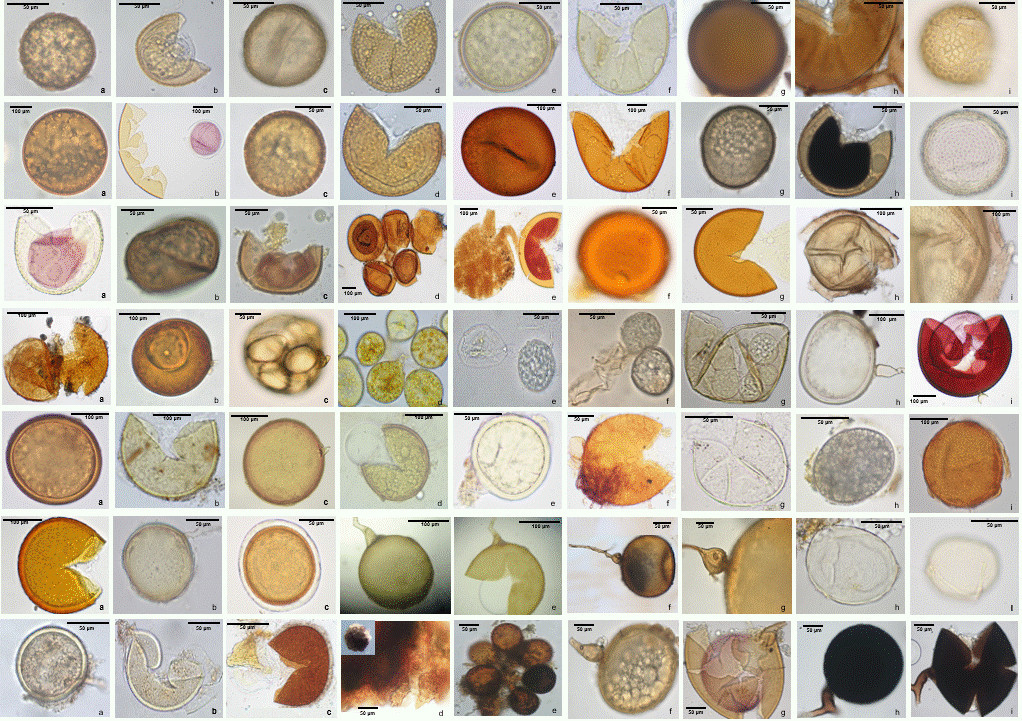
Spores of arbuscular mycorrhizal fungi (AMF), representing the different families within *Glomeromycota* present in Terceira and São Miguel Islands. **Row 1**: a-b) *Ac. brasiliensis*, c-d) *Ac. cavernata*, e-f) *Ac. delicata*, g-h) *Ac. elegans*, i) *Ac. excavata*, h-i); **Row 2**: a-b) *Ac. koskei*, c-d) *Ac. lacunosa*, e-f) *Ac. laevis*, g-h) *Ac. mellea*, i) *Ac. paulinae*; **Row** 3: a) *Ac. paulinae*, b-c) *Ac. spinosa*, d-e) Ac. cf. thomii, f-g) *Ac. tuberculata*, h-i) *Am. appendicula*; **Row 4**: a-b) *Am. fennnica*, c) *Ar. myriocarpa*, d-e) Ar. cf. schenckii, f-g) *Ar. trappei*, h-i) *Ce. pellucida*; **Row 5**: a-b) *Cl. claroideum*, c-d) *Cl. etunicatum*, e) *Cl. lamellosum*, f) *Co. globiferum*, g-h) *Div. celata*, i) *Div. epigaea*; **Row 6**: a) *Div. epigaea*, b) *Div. spurca*, c) *En. infrequens*, d-e) *Fun. mosseae*, f-g) *Gi. margarita*, h) *Par. albidium*, i) *Par. brasilianum*; **Row 7**: a-b) *Rh. clarus*, c) *Sac. baltica*, d) *Scl. sinuosa*, e) *Scl. rubiformis*, f-g) *Scut. calospora*, h-i) *Sept. constrictum*.

Members of the family *Acaulosporaceae* occurred almost exclusively in the native forests, especially associated with the *P. azorica* rhizosphere (Tables 2, 3). The most frequently found members of this family were *Ac. brasiliensis* (48%), followed by *Ac. lacunosa* (26%) and *Ac. mellea* (17%), all exclusively detected in the native forests (Tables 2, 3) (Fig. 3). Five members of this family were only found in pastures, especially in semi-natural areas, including *Ac. excavata*, *Ac. paulinae*, *Ac. thomii*, *Ac. tuberculata* and *Ac. myriocarpa* (Table 2), which may indicate a tendency of some members of this family to occupy less-disturbed habitats (Turrini and Giovannetti 2011, Velázquez et al. 2016) (Fig. 3). Moreover, some AMF species were of restricted distribution. *Ac. spinosa* was detected only in the rhizosphere of *P. azorica* of Serreta (TER_NT_SE); *Ac. thomii* occurred only in the semi-natural pasture of Pico Galhardo (TER_SP_PG); and *Ac. excavata* and *Ac. tuberculata* were both found only in semi-natural pastures of Terra Brava (TER_SP_TB) (Table 2) (Fig. 3). Within the *Glomeraceae*, the AMF species with greater occurrence were *Rhizophagus clarus* (20%), *Sclerocystis rubiformis* (12%) and *Septoglomus constrictus* (10%) (Tables 2, 3) (Fig. 3). The members of this family were distributed throughout all habitats, confirming the great ecological plasticity of *Glomeraceae* members to colonise a broad range of habitats (Öpik et al. 2006) (Tables 2, 3). *Gigasporaceae* was the second most frequent family, being represented by *Scutellospora calospora* (25%) and by *Gi. margarita* (24%) (Tables 2, 3) (Fig. 3). Members of this family also occurred in less disturbed habitats, particularly in the native forests of *P. azorica* (e.g. *Gi. margarita*) (Tables 2, 3) (Fig. 3), although some members of this family were also detected in semi-natural pastures (Table 2). The most frequent members of the family *Archaeosporaceae* was *Ar. myriocarpa* (9%) detected only in pasture systems (Table 2) and *Ar. trappei* (8%) only found in native forests (Tables 2, 3). Within the *Claroideoglomeraceae*, the most frequent species was *Cl. claroideum* (8%), detected only in the native forests (Tables 2, 3) and *Cl. etunicatum* (5%) only found in intensively-managed pastures (Table 2) (Fig. 3).

## Figures and Tables

**Table 1.** Coordinates of sampling sites.

**Site**	**Longitude**	**Latitude**
TER_SP-PG	-27.2232	38.7296
TER_SP_TB	-27.2112	38.7341
TER_IP_R1	-27.1922	38.8296
TER_IP_R2	-27.1950	38.7568
TER_NF_PG	-27.2272	38.7341
TER_NF_LA	-27.3310	38.7496
TER_NF_TB	-27.1979	38.7323
TER_NF_SE	-27.3677	38.7618
SMG_NF_LO	-25.4649	37.7856
SMG_NF_TR	-25.1854	37.7940
SMG_NF_LG	-25.1433	37.7862
SMG_NF_RQ	-25.3020	37.7407

**Table 2.** Species occurrence per habitat type in Terceira Island at four sampling dates (Su – August 2007; Au^1^– September 2012; Sp – May 2013; Au^2^– September 2013): semi-natural pastures (SPPG; SPTB); intensively-managed pastures (IPR1; IPR2) and native forests of *J. brevifolia* (NFPG; NFLA) and *P. azorica* (NFTB; NFSE). (*) Indicates de AMF species, characterised by molecular methods.

	**Terceira**															
**AMF**	**SPPG**	**SPTB**	**IPR1**	**IPR2**	**NFPG**			**NFLA**			**NFTB**			**NFSE**		
	Su	Su	Su	Su	Au^1^	Sp	Au^2^	Au^1^	Sp	Au^2^	Au^1^	Sp	Au^2^	Au^1^	Sp	Au^2^
*Acaulospora brasiliensis* *	-	-	-	-	+	+	+	+	+	+	+	+	+	+	+	+
*Acaulospora cavernata* *	-	-	-	-	-	-	-	-	-	-	+	+	+	-	-	-
*Acaulospora delicata*	+	-	-	-	-	-	-	-	-	-	+	-	-	+	-	-
*Acaulospora elegans*	-	-	+	-	+	-	-	-	-	-	+	+	-	-	-	-
*Acaulospora excavata*	-	+	-	-	-	-	-	-	-	-	-	-	-	-	-	-
*Acaulospora koskei* *	+	-	+	+	+	-	-	-	-	+	+	-	+	-	-	-
*Acaulospora lacunosa* *	-	-	-	-	-	+	+	+	-	-	+	+	+	+	+	+
*Acaulospora laevis* *	+	+	+	+	-	-	-	-	-	-	+		+	-	+	+
*Acaulospora mellea* *	-	-	-	-	-	-	-	-	-	-	-	-	+	+	+	+
*Acaulospora paulinae*	+	+	+	+	-	-	-	-	-	-	-	-	-	-	-	-
*Acaulospora spinosa*	-	-	-	-	-	-	-	-	-	-	-	-	-	+	+	+
*Acaulospora thomii*	+	-	-	-	-	-	-	-	-	-	-	-	-	-	-	-
*Acaulospora tuberculata*	-	+	-	-	-	-	-	-	-	-	-	-	-	-	-	-
*Ambispora appendicula*	-	-	-	-	+	-	-	-	-	-	+	-	-	-	+	+
*Ambispora fennica*	-	-	-	-	-	-	-	-	-	-	-	-	-	-	+	-
*Archaeospora myriocarpa*	+	+	+	+	-	-	-	-	-	-	-	-	-	-	-	-
*Archaeospora schenckii*	+	-	-	-	-	-	-	-	-	-	-	-	-	-	-	-
*Archaeospora trappei*	-	-	-	-	+	-	-	-	-	-	-	-	-	-	-	+
*Cetraspora pellucida*	+	+	+	+	-	-	-	-	-	-	-	-	-	-	-	-
*Claroideoglomus claroideum* *	-	-	-	-	-	-	-	-	-	-	+	+	-	+	-	-
*Claroideoglomus etunicatum*	-	-	+	+	-	-	-	-	-	-	-	-	-	-	-	-
*Claroideoglomus lamellosum*	-	-	-	+	-	-	-	-	-	-	-	-	-	-	-	-
*Corymbiglomus globiferum*	-	-	-	+	-	-	-	-	-	-	-	-	-	-	-	-
*Diversispora celata*	-	-	-	-	+		+	-	-	-	-	+	-	-	-	-
*Diversispora epigaea*	-	-	-	-	-	+	-	-	-	-	-	-	-	-	-	-
*Diversispora spurca*	-	-	-	-	-	+	-	-	-	-	-	-	-	-	-	-
*Entrosphora infrequens*	-	-	-	+	-	-	-	-	-	-	-	-	-	-	-	-
*Funneliformis mosseae*	-	-	-	-	-	-	-	+	+	+	-	-	-	-	-	-
*Gigaspora margarita* *	-	-	-	-	-	-	-	-	-	-	-	+	+	+	+	+
*Paraglomus brasilianum*	-	-	-	+	-	-	-	-	-	-	-	-	-	-	-	-
*Rhizophagus clarus*	+	+	+	+	-	-	-	-	+	+	+	+	+	-	+	-
*Sclerocystis rubiformis*	+	+	+	+	+	+	+	-	-	-	-	+	+	-	+	+
*Sclerocystis sinuosa*	-	-	-	-	-	+	-	-	+	-	-	-	-	-	-	-
*Scuttelospora calospora*	+	+	+	+	-	-	-	-	-	-	+	+	+	+	-	-
*Septoglomus constrictus*	-	-	-	-	-	-	-	-	-	-	-	+	-	-	+	+

**Table 3.** Species occurrence per habitat type in São Miguel Island at three sampling dates (Au^1^ – September 2012; Sp – May 2013; Au^2^ – September 2013): native forests of *J. brevifolia* (NFLO; NFTR) and *P. azorica* (NFLG; NFRQ). (*) Indicates de AMF species, characterised by molecular methods.

	**São Miguel**											
**AMF**	**NFLO**			**NFTR**			**NFLG**			**NFRQ**		
	Au^1^	Sp	Au^2^	Au^1^	Sp	Au^2^	Au^1^	Sp	Au^2^	Au^1^	Sp	Au^2^
*Acaulospora brasiliensis* *	+	+	+	+	+	+	-	-	-	-	-	-
*Acaulospora cavernata* *	-	-	-	-	-	-	-	+	-	-	-	-
*Acaulospora koskei* *	+	-	+	+	+	+	-	-	-	-	-	-
*Acaulospora laevis* *	+	-	-	-	-	-	-	-	-	-	-	-
*Acaulospora mellea* *	-	-	-	-	-	-	+	+	+	+	+	+
*Ambispora appendicula*	-	+	+	-	+	+	-	-	-	-	-	-
*Ambispora fennica*	-	-	-	-	-	-	+	-	-	-	+	-
*Archaeospora trappei*	+	-	-	+	-	-	+	+	+	+	+	+
*Claroideoglomus claroideum* *	-	-	-	+	-	-	+	-	+	+	+	+
*Diversispora celata*	-	+	-	-	-	-	-	-	-	-	+	-
*Diversispora epigaea*	-	+	+	-	-	-	-	-	-	-	-	-
*Gigaspora margarita* *	-	-	-	-	-	-	+	+	+	+	+	+
*Paraglomus albidum*	-	-	-	-	-	-	-	+	-	-	+	-
*Rhizophagus clarus*	-	+	+	+	-	+	-	-	-	-	-	-
*Sacculospora baltica*	-	-	-	-	+	+	-	-	-	-	-	-
*Sclerocystis rubiformis*	+	-	-	+	-	-	-	-	-	-	-	-
*Sclerocystis sinuosa*	-	+	-	-	-	-	-	-	-	-	-	-
*Scuttelospora calospora*	-	-	-	+	+	+	-	-	-	-	-	-
*Septoglomus constrictus*	-	+	+	-	-	-	-	+	+	-	+	+
